# Kyste de la vésicule séminale: à propos d’un cas

**DOI:** 10.11604/pamj.2016.25.68.8240

**Published:** 2016-10-03

**Authors:** Fouad Bakloul, Nabil Jakhlal, Anouar Elghazoui, Tarik Karmouni, Khalid El Khader, Abdellatif Koutani, Ahmed Andaloussi Ibnattya

**Affiliations:** 1 Service d’Urologie B, Hôpital Ibn Sina de Rabat, Maroc

**Keywords:** Kyste, vésicule séminale, agénésie rénale, laparoscopie, Cyst, seminal vesicle, renal agenesis, laparoscopy

## Abstract

Nous rapportons le cas d’un patient présentant un kyste de la vésicule séminale droite symptomatique. Au terme du bilan (échographie pelvienne et endo-rectale, tomodensitométrie abdomino-pelvienne et l’imagerie par résonance magnétique), le patient était opéré et bénéficiait d’une ablation du kyste. A la lumière de cette observation, l’épidémiologie, le diagnostic et les options thérapeutiques sont discutés.

## Abstract (en)

We report the case of a patient with symptomatic seminal vesicle cyst on the right side. Imaging examinations (pelvic and endorectal ultrasound, abdominal-pelvic tomodensitometry and MRI) were perdormed and the patient underwent surgical removal of the cyst. In light of this case study, epidemiology, diagnosis and treatment options are discussed.

## Introduction

Le kyste de la vésicule séminale (KVS) est considéré comme une entité rare, le plus souvent associé à des malformations de développement du canal mésonéphrotique. Le toucher rectal et l’échographie sont la clé du diagnostic. Le traitement des kystes symptomatiques est surtout chirurgical. Nous rapportons l’observation d’un patient qui présente un KVS droit révélé par une hémospermie.

## Patient et observation

**Figure 1:**
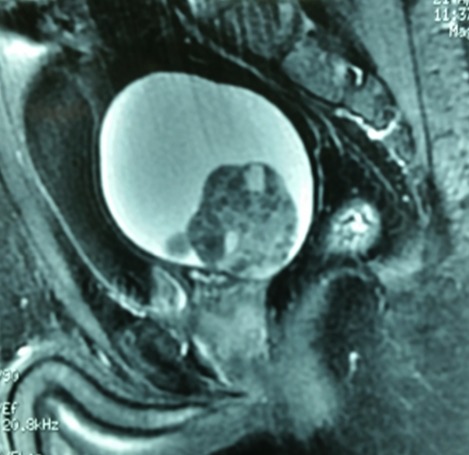
Coupe sagittale de l’IRM montrant un kyste hémorragique de la vésicule séminale, en hypersignal en T1

**Figure 2:**
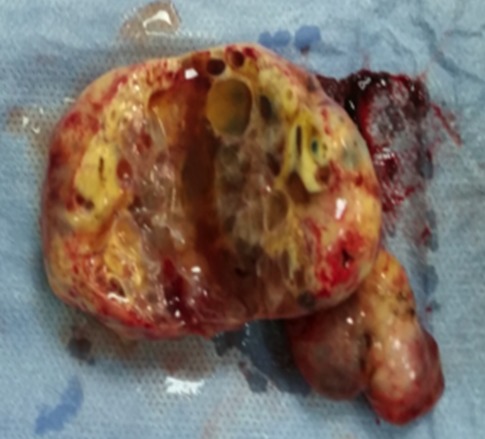
Pièce opératoire du kyste hémorragique de la vésicule séminale

Un homme de 43 ans, ayant antécédent d’infertilité depuis 6 ans, consulte pour une hémospermie intermittente, devenue constante depuis 2 mois, sans autres signes du bas appareil urinaire. L’examen clinique trouve une masse sus-pubienne latéralisée à droite. Le toucher rectal révèle la présence d’une masse fluctuante, pré-rectale et sus prostatique. L’échographie, par voie sus-pubienne et endorectale, objective une volumineuse masse kystique rétro-vésicale, refoulant la vessie en avant et à gauche. La tomodensitométrie confirme le diagnostic d’un KVS droit, mesurant 10×4 cm. L’imagerie par résonance magnétique (IRM) montre un hyper signal en T1 et intermédiaire en T2, ce qui en faveur d’un kyste hémorragique de la vésicule séminale droite (Figure 1). Par ailleurs, on ne met pas en évidence d’anomalie rénale ou urétérale décelable. Le malade était opéré par incision sus-pubienne avec exérèse complète du kyste (Figure 2). Le diagnostic anatomopathologique porté était celui d’un kyste hémorragique de la vésicule séminale droite, confirmant les données de l’IRM. Les suites post-opératoires étaient simples.

## Discussion

Le kyste de la vésicule séminale (KVS) est une affection rare, observé seulement chez 1 sur 20 000 hommes [1]. La plupart de ces kystes s’accompagnent de malformations congénitales secondaires à un défaut de développement du canal méso-néphrotique distal et de l’ébauche urétéral [2]. La malformation la plus fréquente est l’agénésie rénale, suivi par l’abouchement ectopique de l’uretère ipsilatéral. Cet abouchement se fait dans la vésicule séminale elle-même, le canal éjaculateur, le col de la vessie ou l’urètre prostatique. L’uretère est parfois absent. Lorsqu’il est présent, il est borgne [2, 3]. L’association à un spina bifida, à une duplicité urétérale, à l’absence d’un testicule, à une dysplasie rénale controlatérale, à une double veine cave, ou à une absence bilatérale des canaux déférents a également été décrite [3–6]. La majorité des kystes sont de petite taille (<5cm), asymptomatiques et changeant rarement d’évolution au cours du suivi à long terme [7]. Lorsqu’ils sont symptomatiques, les signes cliniques de découverte peuvent être des douleurs périnéales, une éjaculation douloureuse, une hémospermie, une infertilité, [8, 9] des symptômes du bas appareil urinaire [10] ou des infections génito-urinaires [11, 12]. Les kystes volumineux peuvent être palpables dans la région hypogastrique, par contre les petits ne sont accessibles à l’examen physique que par le toucher rectal [13]. L’échographie abdominale permet de situer le kyste par rapport à la vessie et peut identifier les malformations associées du tractus urinaire (agénésie rénale). Elle trouve une image kystique, arrondie ou ovalaire, bien limitée, de siège retro-vésicale. En cas de complication à type d’hémorragie ou d’infection, des échos internes apparaissent [14]. L’échographie transrectale permet une étude anatomique plus détaillée des vésicules séminales et d’identifier d’autres malformations associées des voies séminales. Cet examen peut retenir le diagnostic en confirmant l’origine séminale du kyste [15, 16]. Des méthodes de diagnostic supplémentaires telles que la TDM ou l’IRM sont utilisées lorsque le kyste ne peut être clairement visible sur l’échographie ou en cas de malformations complexes des voies séminales profondes ou anomalies associées du haut appareil urinaire. L’aspect scannographique du KVS est une masse rétro-vésicale, bien définie, de densité liquidienne, qui s’étend de la vésicule séminale à la glande prostatique [2]. L’IRM permet une meilleure analyse objective des voies génitales profondes et complète l’échographie transrectale [17]. Le KVS apparaît généralement en hyposignal sur les séquences T1 et en hypersignal sur T2. L'intensité du signal du kyste que ce soit en T1 ou en T2 varie en fonction du contenu (hémorragie, surinfection, contenu protéique) [2]. La cystoscopie peut aider à confirmer l´absence d´un orifice urétéral en cas d’association d’une agénésie rénale [18]. Le traitement chirurgical des KVS est motivé par la symptomatologie. Plusieurs traitements ont été proposés: la ponction-aspiration et le drainage transurétral ont un risque d’infection pelvienne et de récidive, l’exérèse chirurgicale peut être réalisée par chirurgie ouverte (transvésicale ou transpéritonéale) ou par voie laparoscopique [19, 20]. La laparoscopie transpéritonéale semble être une excellente alternative aux autres voies d’abord, en raison de son caractère mini-invasif et d’une meilleure vision per-opératoire [8, 13]. Mais, la réimplantation urétérale qui s’impose dans certains cas semble être une limite à l’utilisation de cette technique [3].

## Conclusion

Les kystes de la vésicule séminale représentent une pathologie rare, souvent asymptomatiques. Ils sont de plus en plus de découverte fréquente grâce au développement des moyens d’imagerie. Le traitement des kystes symptomatiques reste principalement chirurgical.
